# Combination of *Anoectochilus roxburghii* Polysaccharide and Exercise Ameliorates Diet-Induced Metabolic Disorders in Obese Mice

**DOI:** 10.3389/fnut.2021.735501

**Published:** 2021-10-08

**Authors:** Cong Chen, Meisong Kang, Qiaowen Wang, Weilin Liu, Minguang Yang, Shengxiang Liang, Qing Xiang, Xiao Han, Jing Tao

**Affiliations:** ^1^The Institute of Rehabilitation Industry, Fujian University of Traditional Chinese Medicine, Fuzhou, China; ^2^College of Biological Science and Engineering, Fuzhou University, Fuzhou, China

**Keywords:** *Anoectochilus roxburghii* polysaccharide, exercise, metabolic disorders, type 2 diabetes, oxidative stress

## Abstract

Metabolic syndrome is a cluster of metabolic disorders that threatens public health. Nevertheless, its exact mechanism and relative intervention remain largely obscure. Accumulating evidence indicate that tither *Anoectochilus roxburghii* polysaccharide (ARP) or exercise (EX) exhibited the beneficial effects on metabolic health. However, the synergetic beneficial effects of ARP and EX as a combined intervention on obesity-induced metabolic disorders remain largely obscure. Male C57BL/6 mice were fed a high-fat diet (HFD) and intervened with ARP and EX for 12 continuous weeks. The results indicated that the ARP, EX, and ARP combined with EX treatment group regulated lipogenesis by suppressing the fatty acid pathway, dampening the system oxidative stress by stimulating Nrf2-mediated phase II enzyme system, and promoting the mitochondrial function by activating the mitochondrial complexes and PGC-1α in HFD mice. More importantly, the combination of ARP and EX showed an even greater beneficial effects relative to either ARP or EX alone, especially in decreasing reactive oxygen species (ROS) level and increasing adenosine triphosphate (ATP) content. Taken together, these findings further confirmed that ARP and EX could be effective interventions on obesity-induced metabolic abnormalities, and that the combination of ARP and EX exhibited the beneficial synergetic effects.

## Introduction

The metabolic syndrome, characterized by obesity and its related metabolic diseases, has become a major public health concern in modern society (1). The pathologic manifestation of metabolic syndrome (MetS) presents as abdominal obesity, impaired glucose tolerance, dyslipidemia, insulin resistance, and hypertension (2). In clinics, hyperlipidemia, described as excessive lipid deposition in body, has been considered to be a central and causal risk factor for MetS (3, 4). Excessive exposure to high-fat diet (HFD), long-term stress, and lack of exercise observed in unhealthy lifestyles would increase energy accumulation in the body, which is the primary cause of hyperlipidemia (5). In addition, substantial evidence has indicated that excessive lipids stimulated the abnormalities of cytokines, including tumor necrosis factor-α (TNF-α) (6), interleukin-6 (IL-6), (7), and leptin (8), which gave rise to a vicious process that leads to fat deposition, and thus participates in the pathogenesis of obesity-associated MetS. Although the exact pathological mechanism underlying the association between MetS and hyperlipidemia remains largely obscure, available reports evidently demonstrated that adipocyte abnormalities contribute to the progression of MetS (9). Emerging evidence reveal that excessive lipid is closely related to the onset of metabolic-induced diseases, including diabetes, cardiovascular diseases, and certain form of cancers (10, 11).

Further, metabolic disorder has been proved to be strongly related to the system oxidative stress, and therefore affects metabolic state (12, 13). The clinical research indicated that obese subjects exhibit a higher oxidative stress in body (14). Animal studies also found that mice fed with HFD can result in the elevated level of reactive oxygen species (ROS) in livers, which preceded the manifestation of obesity (15, 16). Other than the activation of oxidative stress, MetS has been recognized to be strongly associated to mitochondrial function, activity, dynamics and turnover (17, 18). With the globalization of obesity and its related MetS, growing attention has been drawn to the exploitation of pharmacological therapy.

Currently, dietary modifications and physical exercise (EX) intervention are still considered as clinically beneficial mainstays to promote metabolic health (19, 20). To date, many natural extracts have been demonstrated to exhibit effective effects on the progression of MetS. *Anoectochilus roxburghii* is a classical traditional Chinese herb and called “king's medicine” in southern China (21). Owing to its minimal toxicity and therapeutic efficacy, *A. roxburghii* has gained interest for centuries due to its hepatoprotective, anti-diabetes, anti-inflammatory, antitumor, and antioxidant effects (22–25). Some recent studies revealed that *A. roxburghii* polysaccharose (ARP) has been attributed with antidiabetic effects in the improvement of glucose and lipid metabolism (22, 26, 27). However, whether ARP combined with EX regulates obesity-induced metabolic disorders has not been investigated *in vivo*. Meanwhile, how this combination intervention exerts its protective effect remains largely unexamined. Herein, it is of great significance to illustrate its regulatory mechanism.

The purpose of the present study was to investigate the synergetic effects of ARP combined with EX in C57BL/6 HFD-fed mice, and to further explore the detailed mechanism underlying the protective effects on obesity-induced metabolic disorders. The findings would unveil a better understanding of the combination intervention in improving metabolic health and find more potential therapy targets.

## Materials and Methods

### Chemicals

Primary antibodies against NF-E2 related factor (Nrf2, #sc-365949), heme oxygenase-1 (HO-1, #sc-390991) and NAD(P)H/quinone oxidoreductase (NQO1, #sc-376023) were obtained from Santa Cruz Biotechnology (Dallas, TX, USA). Antibodies against fatty acid synthase (FAS, #3180), acetyl-coenzyme A carboxylase 1 (ACC1, #4190), peroxisome proliferator-activated receptor gamma coactivator-1α (PGC-1α, #2178) and β-actin (#58169) were purchased from Cell Signaling Technology (Danvers, MA, USA). Antibodies against NDUFS3 (#459130), SDHB (#459230), UQCRC1 (#459140), MTCO1 (#459600), and ATP5A1 (459240) were acquired from Thermo Fisher Scientific (Waltham, MA, USA).

### Extraction and Characterization of *A. roxburghii* Polysaccharose

*A. roxburghii* was purchased from the Drug Store Pharmacy of Fuzhou (Fuzhou, China). To examine the optimum conditions for the extraction of *A. roxburghii* polysaccharose (ARP), the influence of various factors were tested, including ultrasonic temperature, power, time, and material liquid ratio. Briefly, dried *A. roxburghii* was exhaustively extracted in distilled water according to the liquid-to-material ratio of 1:3 (v/v), followed by an ultrasound at 200 W for 30 min, and then supplemented with distilled water with the ratio of 1:35 (v/v) at 80°C for 2 h. Next, the solution was centrifuged and concentrated with a rotary evaporator at 80°C. Then, the concentration was deproteinated with a Sevag method, washed with acetone and ether, and finally precipitated with EtOH for purification. The average yield of ARP is ~3.16% (w/w). For ARP analysis, the phenol-sulfuric acid method (with glucose as the standard) was performed to measure the degree of the purity of ARP (28). One milliliter (1 ml) phenol solution (50 g/L) was injected into 1 ml glucose solution with different concentrations (0, 20, 40, 60, 80, and 100 mg/L), and 5 ml concentrated sulfuric acid was added for 30 min. After incubation, absorbance was measured to be at 490 nm by the microscope. Subsequently, the ARP content was further examined according to the standard curve.

### Acute Toxicity Study

According to the previous studies (27), the acute toxicity effect of ARP was evaluated for its safety. The ARP at 100, 200, and 400 mg/kg dose concentration has been tested for the treated mice. No significant difference of toxicity and mortality has been observed between ARP treatment and control group. Taken together, a 400 mg/kg ARP treatment concentration was used for the following assays.

### Animal and Experimental Design

Male C57BL/6 mice (SPF, 4-week-old) in this study were purchased from the Shanghai Laboratory Animals Center (SLAC). Mice were kept under a condition of 24 ± 0.5°C, relative humidity of 60% with a 12 h light/dark cycle. After 1 week of adaptation, the mice have been randomly divided to five groups (*n* = 8 per group), namely, normal-food diet (control, 10 kcal%, Cat #D12492, Research Diets, New Brunswick, NJ), high-fat diet (HFD, 60 kcal%, Cat #D12450, Research Diets), HFD plus daily administration of 400 mg/kg/day ARP (HFD+ARP), HFD plus exercised (HFD+EX, 12 m/min for 30 min at 5 days per week), and HFD plus daily administration of ARP plus exercised (HFD+ARP+EX). All the administrations were conducted between 9 and 10 o'clock in the morning, and body weight was measured every week. All animal experiments were approved by the Animal Ethics Committee in Fujian University of Traditional Chinese Medicine and performed according to the guidelines of animal care and use for scientific purposes.

### Treadmill Exercise

Mice were adapted on treadmill for 5 consecutive days before pre-exercise training. All mice in EX groups commit exercise training five times every week for 12 continuous weeks. The exercise program was based on a previous study (29). In brief, from the 1st to 4th week, all mice in EX groups were made to do a mild intensity run on a treadmill at a speed of 5 m/min for the first 5 min, at a speed of 10 m/min for the next 30 min, and further at a speed of 5 m/min for the last 5 min. From the 5th to the 12th week, mice commit a moderate intensity at 5/min for the first 5 min, then 13 m/min for the next 30 min, and further at 5 m/min for the last 5 min.

### Oral Glucose Tolerance Test (OGTT)

At the 12th week of intervention, an OGTT was further examined. After the mice were fasted overnight, the glucose was administrated (1 g/kg body weight) in the mice. Blood was taken from the tail vein every 30 min for over 2 h, and the concentration of plasma glucose was finally examined by an Accu-Chek meter (Roche, Basel, Switzerland).

### Samples Preparation

After mice were sacrificed, small portions of liver tissues were quickly excised, weighed, homogenized in chilled PBS, and the supernatant was separated by centrifugation (12,000 rpm, 30 min). Meanwhile, blood samples were collected by cardiac puncture, and the serum was obtained with centrifugation (3,500 rpm, 20 min). The tissue and serum samples were prepared for the following experiment.

### Biochemical Parameters Analysis

The concentrations of total cholesterol (TC), triacylglycerol (TG), low-density lipoprotein cholesterol (LDL), high-density lipoprotein cholesterol (HDL), superoxide dismutase (SOD), malondialdehyde (MDA), glutathione peroxidase (GPX), reduced glutathione (GSH), glutathione S-transferase (GST) and glucose in serum or liver homogenates were examined by biochemical kits (Nanjing Jiancheng, China). The content of insulin, tumor necrosis factor-α (TNF-α), C-reactive protein (CRP), and interleukin-6 (IL-6) was analyzed by enzyme-linked immunoassay (ELISA) kits (Nanjing Jiancheng). The content of ATP was examined with bioluminescent kits (Sigma, MO, USA).

### Histological Observation

Liver tissues were removed, rinsed with chilled phosphate-buffered saline (PBS), then fixed in 10% paraformaldehyde solution. The treated-tissues have been embedded in paraffin, sectioned (thickness 4–5 μm), stained with hematoxylin and eosin (H&E), and visualized by Olympus BX71 microscope.

### Protein Carbonylation Assessment

The level of protein carbonyls was examined by the Oxyblot protein oxidative detection kit (Cell Biolabs, San Diego, CA). The content of protein carbonyls was determined using western blot assay, and the sample amount was measured using Coomassie brilliant blue.

### Reactive Oxygen Species (ROS) Measurement

The level of intracellular ROS has been measured using a fluorescence with 2'7'-dichlorofluorescein (DCF-DA). The fluorescence wavelength with 488 nm excitation and 535 nm emission was used for detection by a microplate fluorometer.

### Western Blot

The protein sample was extracted using immunoprecipitation (IP) lysis buffer, and then concentrated by the bicinchoninic assay (BCA) kit (Beyotime, China). The equal amounts of samples were injected in an 10% sodium dodecyl sulfate polyacrylamide gel electrophoresis (SDS-PAGE), and further transferred onto a hydrophilic polyvinylidene fluoride (PVDF) membrane. The membrane was blocked with 5% non-fat-milk and then incubated with the appropriate antibodies. In another day, the membrane was incubated with the secondary antibodies. Finally, the protein band was visualized by an enhanced chemiluminescence (ECL) plus reagent.

### Statistical Analysis

All the data in the study were exhibited as mean ± standard error of the mean (S.E.M). The one-way ANOVA was analyzed by GraphPad Prism 8.0 (San Diego, CA) for statistics. For analysis, *p* < 0.05 was utilized as statistically significant.

## Results

### ARP and EX Reduced Body and Tissue Weights in HFD-Fed Mice

**Figure 1:**
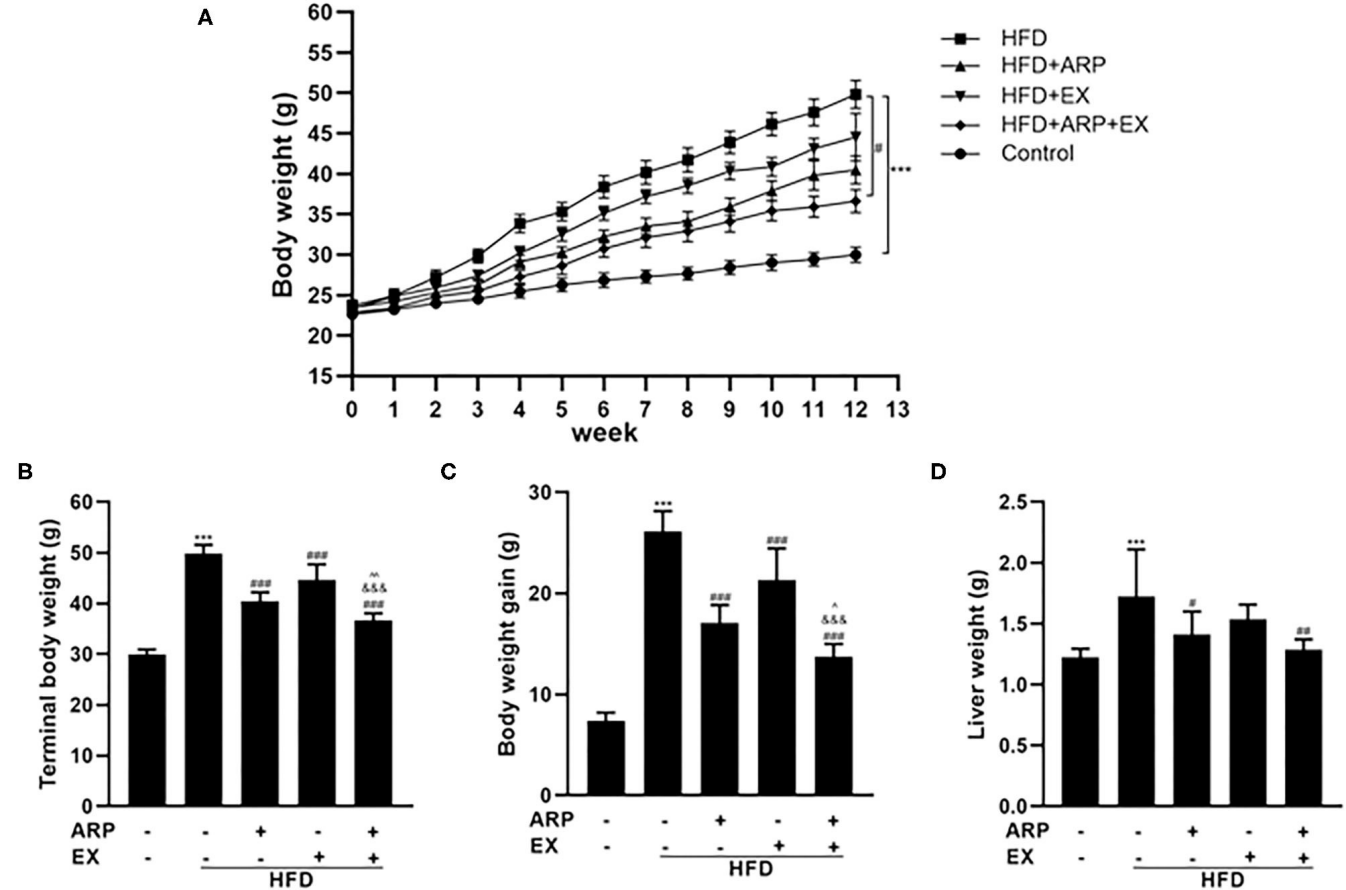
Effects of *Anoectochilus roxburghii* polysaccharide (ARP) and exercise (EX) on body and tissue weights in high fat diet (HFD)-fed mice. **(A)** The curve of body weight, **(B)** terminal body weight, **(C)** body weight gain, **(D)** liver weight in C57BL/6 mice feeding HFD. All the values are exhibited as the mean ± SME, *n* = 8. ***p < 0.001 vs. related control group. ^#^*p* < 0.05, ^##^*p* < 0.01, ^###^*p* < 0.001 vs. related HFD group; ^∧^*p* < 0.05, ^∧∧^*p* < 0.01 vs. related HFD+AR group; ^&&&^*p* < 0.001 vs. related HFD+EX group.

The high-fat diet C57BL/6 mouse is a universal used animal model for the study of obesity-associated diabetes. Being overweight or obese is believed to accelerate the risk of developing MetS (30). In the present study, mice have been randomly assigned into 5 groups at the beginning of the diet and ARP and EX intervention. The average body weight at initial among the five groups showed no significant difference. Subsequently, the body weight in each group continuously increased (Figure 1A). During the continuous 12 weeks of feeding, the body weight in the HFD group showed a significantly higher weight relative to the normal diet mice. Compared with the HFD group, the body weight of the HFD+ARP, HFD+EX and HFD+ARP+EX groups decreased, but only the HFD+ARP+EX group showed a statistical significance. The terminal body weight and body weight gains showed a 66.7 and 254.1% increase in the HFD group as compared with the normal group and significantly decreased in the ARP and EX treatment groups (Figures 1B,C). Compared to either HFD+ARP or HFD+EX group, the HFD+ARP+EX significantly decreased in the final body weight and body weight gain. At the end of 12 weeks, the liver weight was examined. The liver weight in the HFD group showed a 40.8% increase than that of the normal group, while ARP and EX intervention showed a significant decrease than that of the HFD group (Figure 1D). Compared to the HFD group, the liver weight of the HFD+ARP, HFD+EX, and HFD+ARP+EX group reduced by 18.1, 10.7, and 25.4%, respectively. The results indicated that ARP and EX can reduce the body and liver weight in HFD-fed mice.

### ARP and EX Ameliorated HFD-Induced Insulin Sensitivity in Mice

**Figure 2:**
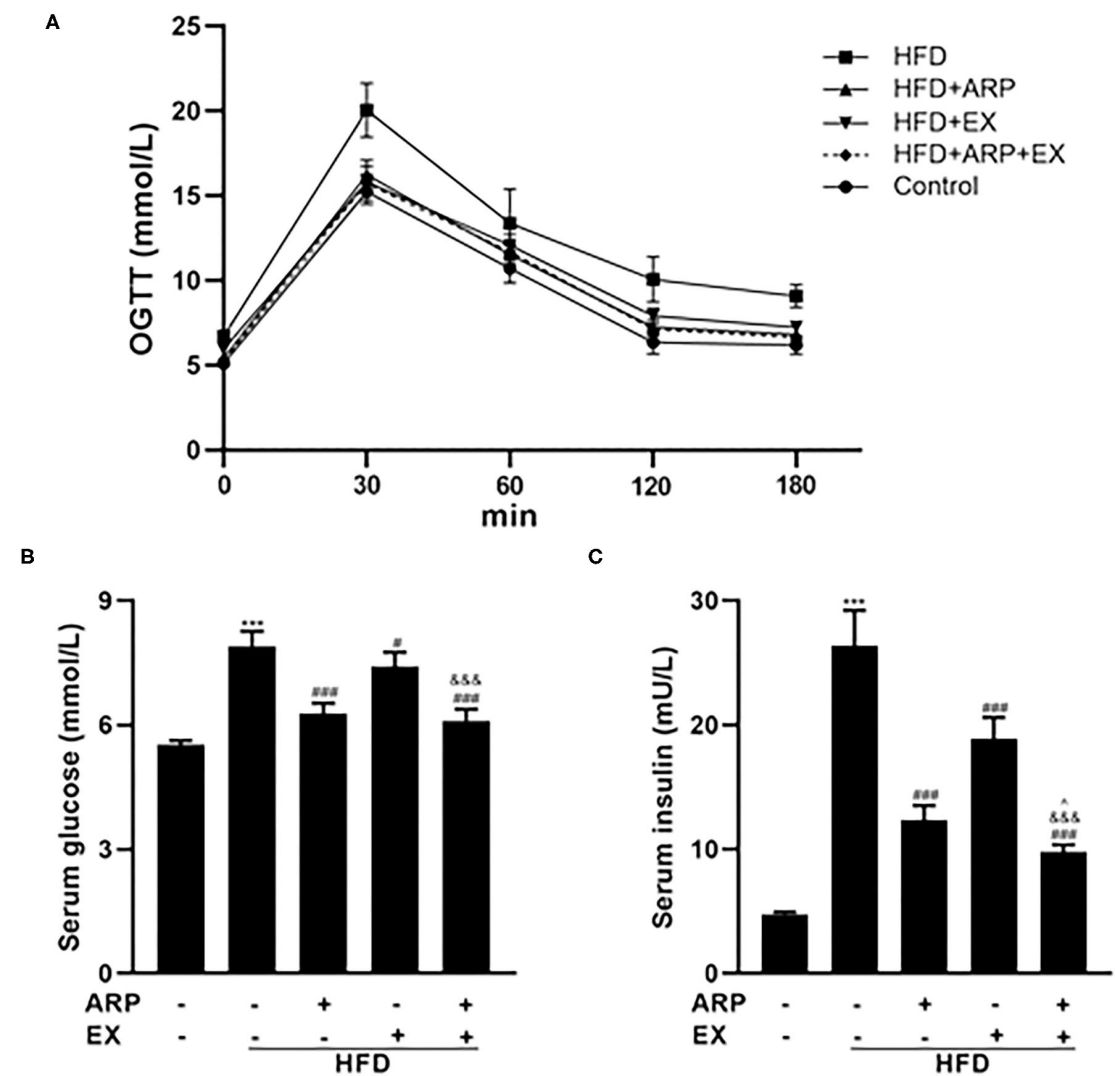
Effects of ARP and EX on insulin sensitivity in HFD-fed mice. **(A)** Blood glucose tolerance, **(B)** serum glucose, **(C)** serum insulin in C57BL/6 mice feeding HFD. All the values are exhibited as the mean ± SME, *n* = 8. ***p < 0.001 vs. related control group. ^#^*p* < 0.05 and ^###^*p* < 0.001 vs. related HFD group; ^∧^*p* < 0.05 vs. related HFD+AR group; ^&&&^*p* < 0.001 vs. related HFD+EX group.

At the 12th week, an OGTT was detected and revealed that the insulin sensitivity showed an obvious decrease in the HFD group relative to the normal control group, which was increased in the ARP and EX treatment group (Figure 2A). The serum glucose concentrations in the HFD+ARP, HFD+EX, and HFD+ARP+EX groups exhibited a markedly lower level than that in the HFD group to a 20.5, 6.2, and 22.7% decrease, respectively (Figure 2B). Similar results were observed with serum inulin contents.

The HFD+ARP, HFD+EX, and HFD+ARP+EX showed a 53.2, 28.4, and 62.9% decrease as compared to the HFD group, suggesting potential insulin resistance (Figure 2C). More importantly, the combined ARP and EX treatment group displayed a significant decrease of hypoglycemia than either the ARP or the EX group.

### ARP and EX Influenced HFD-Induced Serum Cytokines in Mice

**Figure 3:**
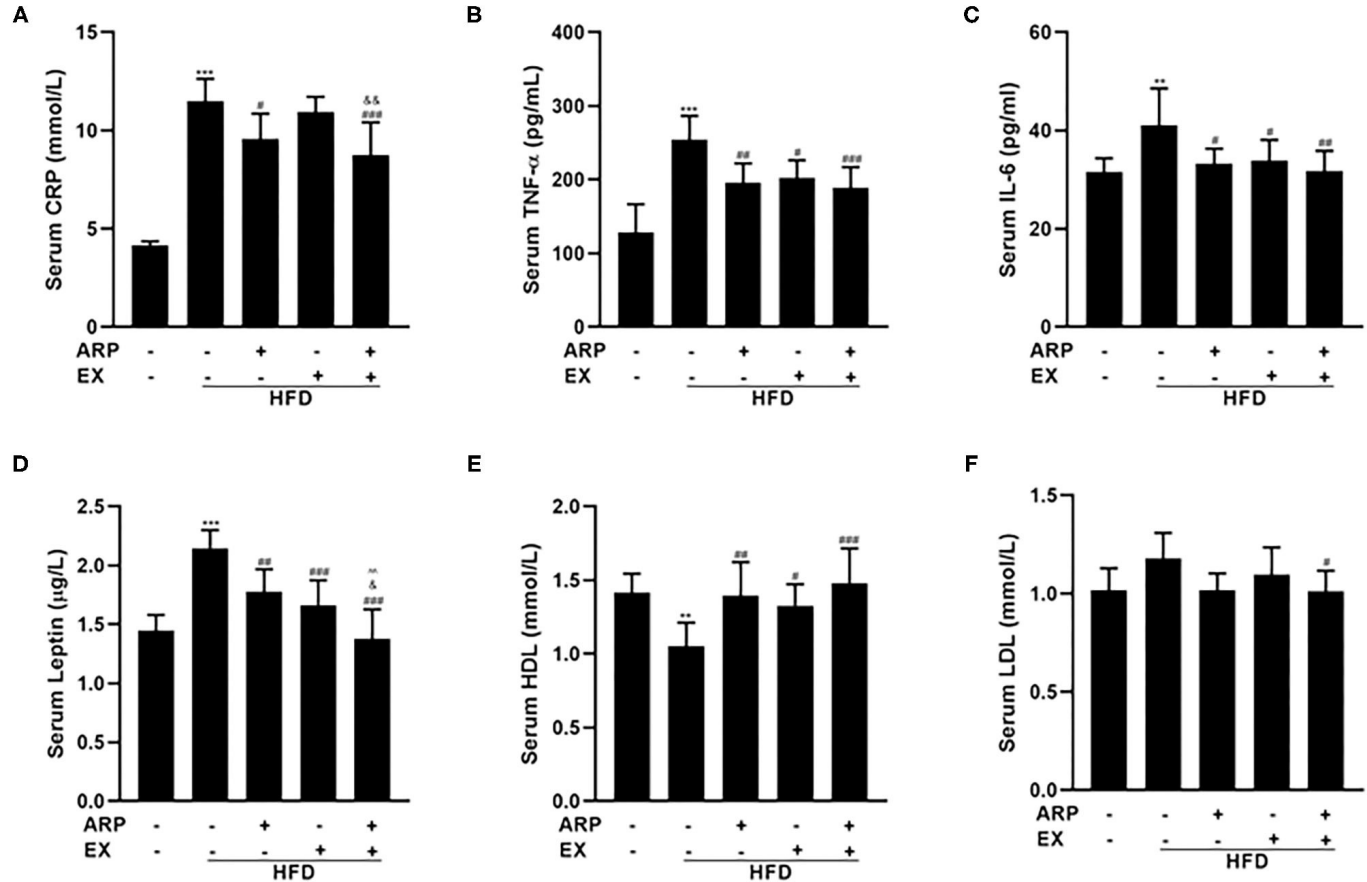
Effects of ARP and EX on serum cytokines in HFD-fed mice. Serum contents of CRP **(A)**, TNF-α **(B)**, IL-6 **(C)**, leptin **(D)**, HDL **(E)**, and LDL **(F)** in HFD-fed mice treated with ARP and EX. All the values are exhibited as the mean ± SME, *n* = 8. **p < 0.01 and ***p < 0.001 vs. related control group. ^#^*p* < 0.05, ^##^*p* < 0.01, ^###^*p* < 0.001 vs. related HFD group; ^∧∧^*p* < 0.01 vs. related HFD+AR group; ^&^*p* < 0.05, ^&&^*p* < 0.01 vs. related HFD+EX group.

Inflammation has emerged as a critical factor in the main cause of T2DM complications as it may trigger the onset and progression of diabetes (31). As expected, the HFD-fed mice showed a 170.8% increasing serum CRP concentration, a liver-secreted cytokine in modulating inflammatory responses (32, 33). The HFD+ARP and HFD+ARP+EX groups could effectively attenuate CRP levels and exhibit a 16.7 and 23.8% decrease, but HFD+EX had no obvious effect on CRP (Figure 3A). In addition, serum levels of pro-inflammatory cytokines have been strongly associated with MetS (34). As illustrated in Figures 3B,C, the serum levels of TNF-α and IL-6 in the HFD group were remarkedly elevated relative to the normal control group. Additionally, the administration of ARP and EX treatment sufficiently attenuated these levels. Long-term HFD feeding would cause a decrease in the sensitivity of central leptin (35). A 48.3% elevated leptin content was found in the HFD group (Figure 3D), and these serum cytokines levels were successfully diminished in HFD+ARP, HFD+EX, and HFD+ARP+EX groups by 17.2, 22.8, and 35.8%, respectively, as compared with the HFD-fed group. More importantly, the leptin content in HFD+ARP+EX group showed a more significant decrease than that in either HFD+ARP or HFD+EX group. In addition, HDL, and LDL, which are usually abnormal in HFD-fed mice, were further examined. The HDL level showed a 26.1% decrease in the HFD group, while ARP or EX or combined ARP and EX treatment alleviated this abnormality (Figure 3E). The LDL level was increased in HFD group, and only combined ARP and EX treatment sufficiently reversed this effect (Figure 3F). These results suggested that ARP and EX not only alleviated the inflammatory responses, but also reduced blood lipid.

### ARP and EX Reduced Liver Lipid Accumulation in HFD-Fed Mice

**Figure 4:**
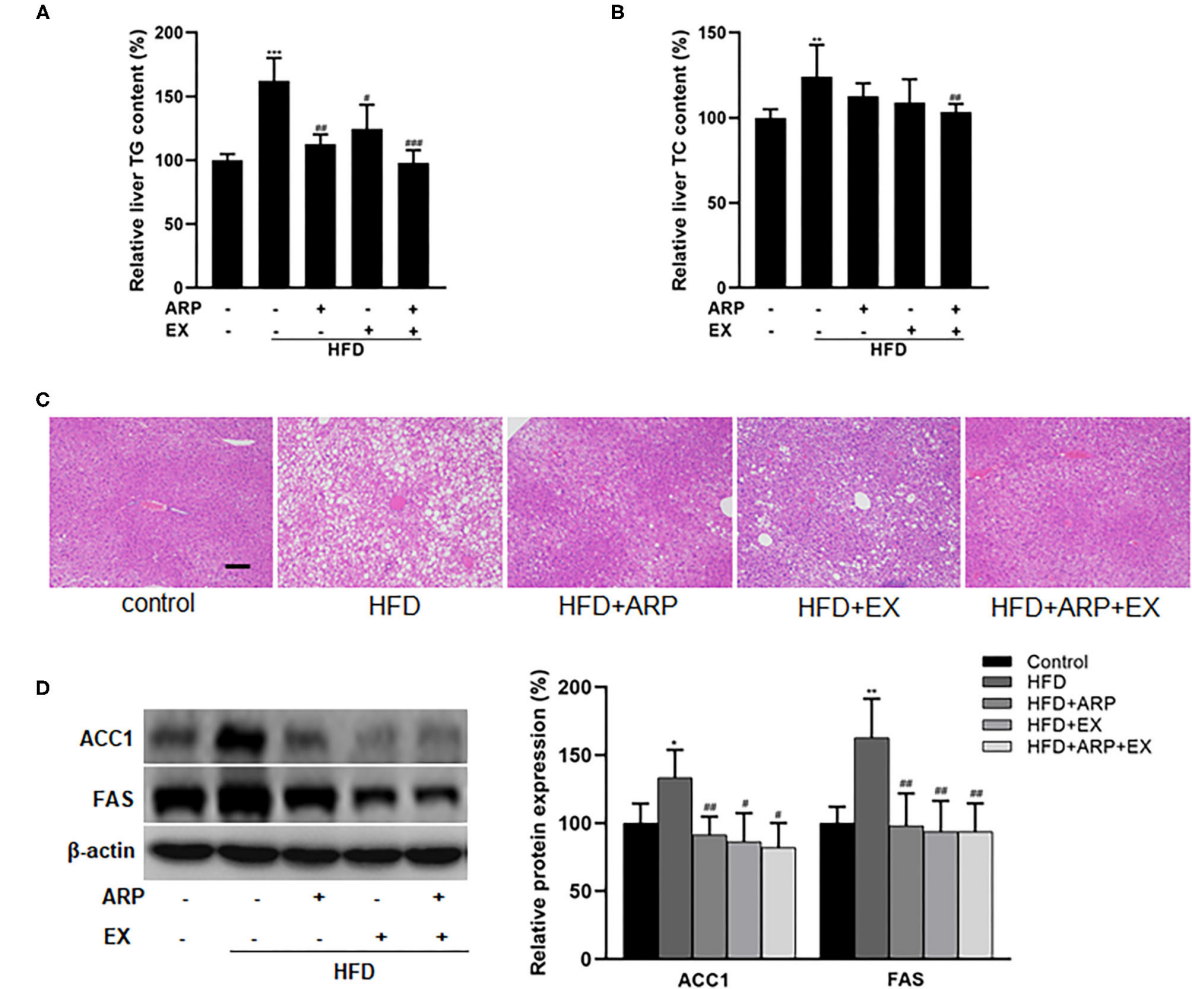
Effects of ARP and EX on lipid accumulation in HFD-fed mice. **(A)** Liver TG content. **(B)** Liver TC contents. **(C)** The histological images of H&E staining in liver tissues. **(D)** Liver expression of FAS and ACC1 (left, western blot image; right, statistical analysis). TC, total cholesterol; TG, tri-glycerides. H&E staining scale bars: 100 μm. All the values are exhibited as mean ± SME, *n* = 8. *p < 0.05, **p < 0.01, and ***p < 0.001 vs. related control group. ^#^*p* < 0.05, ^##^*p* < 0.01, ^###^*p* < 0.001 vs. related HFD group.

Hyperlipidemia is usually manifested as abnormal lipid metabolism (4). The HFD group significantly increased liver TG and TC levels, whereas ARP or EX or combined ARP and EX treatment sufficiently attenuated the lipid profiles, as evidenced by significant reductions in the lab test results. For liver TG level, the HFD-fed showed a 62.3% increase as compared with the normal control group, while the HFD+ARP, HFD+EX, and HFD+ARP+EX groups decreased the abnormality in various degrees as 30.7, 23.3, and 39.6%, respectively (Figure 4A). For liver TC level, the HFD group exhibited a 24.1% increase in contrast to the normal group, and only the HFD+ARP+EX group could reduce its TC content (Figure 4B). In addition, H&E staining has been applied to display liver lipid deposits. The HFD group presented an obvious lipid accumulation, and other three interventive groups markedly ameliorated this appearance caused by HFD intervention (Figure 4C). Meanwhile, the effects of ARP and EX on critical regulators of fatty acid synthesis were investigated. The protein expression of liver ACC1 and FAS in the HFD group showed a 33.5 and 62.8% increase compared with the normal control group, whereas ARP or EX or combined ARP and EX treatment exhibited significantly decreased in these two lipogenic genes (Figure 4D). Taken together, these results demonstrated that ARP and EX treatment could prevent HFD-induced fat accumulation by inhibiting the fatty acid pathway.

### ARP and EX Attenuated Liver Oxidative Stress in HFD-Fed Mice

**Figure 5:**
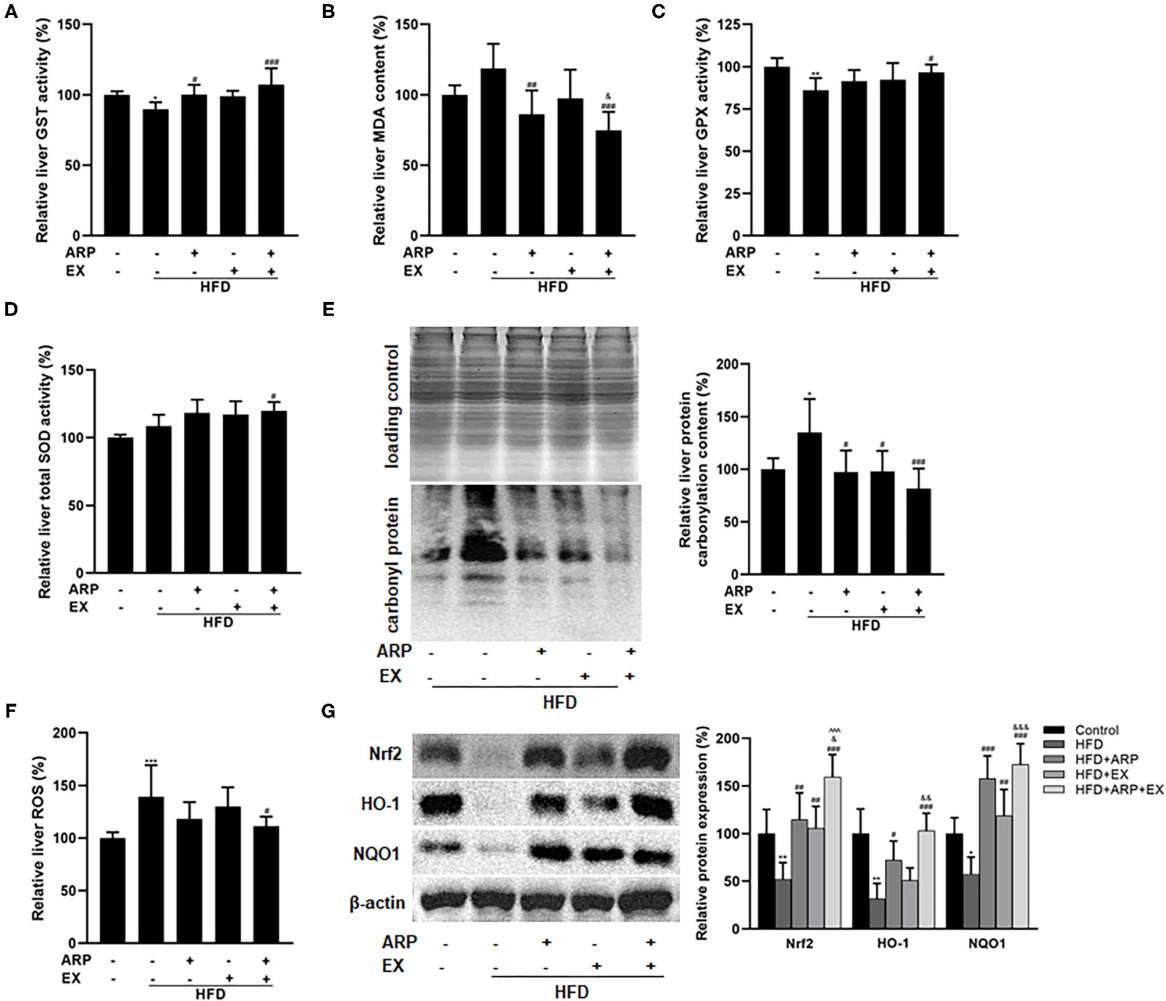
Effects of ARP and EX on liver oxidative stress in HFD-fed mice. **(A)** Liver GST activity. **(B)** Liver MDA content. **(C)** Liver GPX activity. **(D)** Liver SOD activity. **(E)** Liver protein carbonyl content (left, western blot image; right, statistical analysis). **(F)** Liver ROS level. **(G)** Liver expression of Nrf2, HO-1 and NQO1 (left, western blot image; right, statistical analysis). All the values are exhibited as the mean ± SME, *n* = 8. *p < 0.05, **p < 0.01 and ***p < 0.001 vs. related control group. ^#^*p* < 0.05, ^##^*p* < 0.01, ^###^*p* < 0.001 vs. related HFD group; ^∧∧∧^*p* < 0.001 vs. related HFD+AR group; ^&^*p* < 0.05, ^&&^*p* < 0.01, ^&&&^*p* < 0.001 vs. related HFD+EX group.

Oxidative stress, an imbalance between oxidants and antioxidants, has been considered as a critical contributor in the pathogenesis of obesity-associated disease (36). Relative to the HFD group, the administration of ARP (HFD+ARP and HFD+ARP+EX groups) effectively elevated the GST activity and reduced the MDA formation (Figures 5A,B). The combined ARP and EX treatment was the only group that successfully promoted 12.3% in the total SOD activity and 10.3% in the GPX activity, whereas either ARP or EX had no significant effect (Figures 5C,D), suggesting a significant synergetic effect of ARP and EX. Subsequently, carbonyl protein amounts were measured to evaluate the protein oxidative status. The carbonyl protein contents exhibited a 34.8% increase in the HFD group, and the ARP and EX intermediate groups inhibited the varying increased level of protein carbonyl. More importantly, combined ARP and EX group sufficiently attenuated the effect to a greater extent (39.4%) than either the ARP (27.8%) or EX group (26.8%) (Figure 5E). In addition, excessive ROS can cause severe liver oxidative damage. The result showed that the ROS content showed a 39.3% increase in the HFD group, while only the HFD+ARP+EX group would prevent this elative status and exhibit a 20.0% decrease (Figure 5F). In response to oxidative stress, one of the antioxidant systems, phase II enzyme, is usually activated to defense the oxidative stress. The protein contents of Nrf2 and its target genes, HO-1 and NOQ1, were further measured. As illustrated in Figure 5G, the HFD group significantly decreased the Nrf2 (68.3%), HO-1 (68.3%), and NQO1 (42.5%) expression, whereas ARP or EX or combined ARP and EX treatment significantly increased all three protein contents. Among them, the HFD+ARP+EX group showed a greater elevation than the other two treatment groups. These results indicated that ARP and EX treatment could sufficiently alleviate HFD-induced oxidative damage by upregulating phase II enzymes.

### ARP and EX Improved Mitochondrial Biogenesis in HFD-Fed Mice

**Figure 6:**
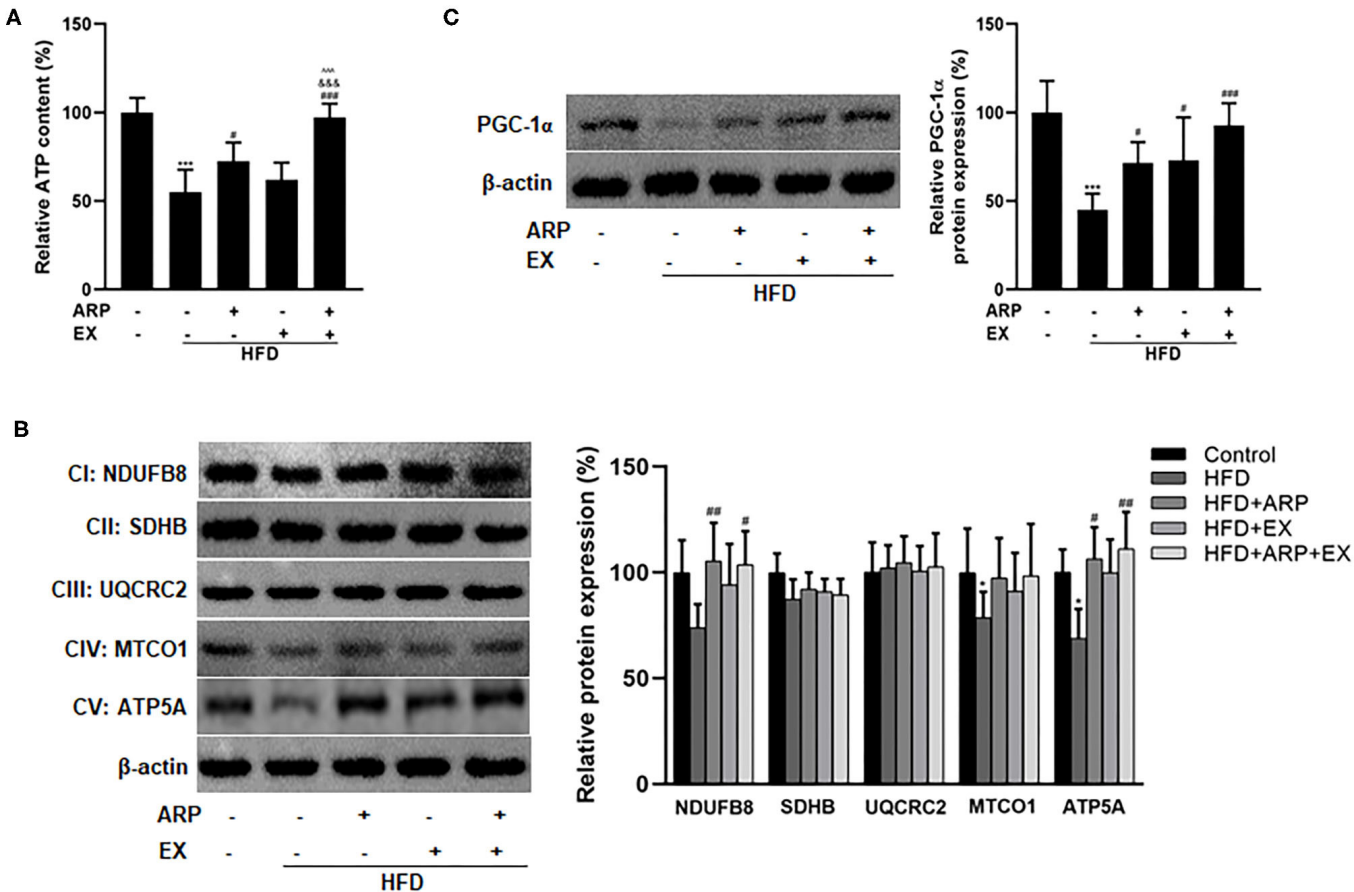
Effects of ARP and EX mitochondrial biogenesis in HFD-fed mice. **(A)** Liver ATP content. Liver expression of PGC-1α **(B)** and mitochondrial complexes subunits **(C)** (left, western blot image; right, statistical analysis). All the values are exhibited as mean ± SME, *n* = 8. *p < 0.05 and ***p < 0.001 vs. related control group; ^#^*p* < 0.05, ^*##*^*p* < 0.01, and ^*###*^*p* < 0.001 vs. related HFD group; ^∧∧∧^*p* < 0.001 vs. related HFD+AR group; ^&&&^*p* < 0.001 vs. related HFD+EX group.

Mitochondria are the cytoplasmic organelles strongly associated with cellular energy metabolism (37). To explore the involvement of mitochondria in HFD-fed mice, the liver ATP homeostasis was further examined. The results showed that the depletion of ATP contents reducing a 45% extent was triggered by HFD intervention, while ARP and combined ARP+EX treatment obviously promoted the ATP contents to a 31.8 and 76.9% increase, respectively. Obviously, combined ARP and EX treatment efficiently reversed the HFD-induced oxidative stress (Figure 6A). In addition, the results found the downregulated protein expression of mitochondrial complexes IV and V in the liver HFD-fed mice having a 21.4 and 31.1% decrease as compared with the normal control group, suggesting an impairment in mitochondrial respiratory chain. However, the HFD+ARP and HFD+ARP+EX treatment could only effectively improved complexes V activity to a 54.5 and 61.2% increase, respectively (Figure 6B). Furthermore, the PGC-1α expression, a well-known marker of mitochondrial oxidative metabolism, was examined. The results indicated that the HFD+ARP, HFD+EX, and HFD+ARP+EX treatment markedly upregulated the PGC-1α protein level as compared with the HFD group to a 58.9, 62.2, and 105.4% elevation, respectively (Figure 6C).

## Discussion

Due to unhealthy dietary lifestyle, the prevalence of MetS has reached global epidemic proportions. As a critical risk factor of MetS, obesity is one of the most common lipid abnormalities and has been proved to be strongly associated with metabolic-related disorders, including diabetes, cardiovascular and fat liver diseases (38–40). The current therapeutic approach that involves weight loss and metabolic pathologies is not satisfactory. Recent findings have evidenced that nutritional combined with physical EX interventions may be a more effective strategy than diet alone on improving the progression of MetS (19, 39). The objective of the study was to explore the beneficial effect of ARP and EX on obesity-associated metabolic disorders.

It is well-established that MetS is accompanied by an increased risk of hyperlipidemia, and the excessive fat or lipid significantly deposited under this state (41). Excessive energy that exceeds the body's ability to store fat in adipose tissue as triglycerides, circulates lipids from various sources and spill over into non-adipose tissues, which then increases hypertrophy and hyperplasia of adipose tissues, and eventually leads to metabolic disorders (42). Previous studies have shown that either ARP or EX could efficiently reduce the body weight and lipids in obese mice (43–46). In agreement with them, the present study found that ARP, EX, or ARP+EX group had the reductive effects on the body mass and liver lipids accumulation in HFD-induced obesity mice. More importantly, the body mass in the HFD+ARP+EX group exhibited a greater decrease than that in the HFD+ARP and HFD+EX groups, suggesting that the combination of ARP and EX exerted a more efficient effect on the body weight.

Previous studies have revealed that the MetS disorder is closely associated with excessive lipid accumulation. In the study, it was discovered that the liver contents of TG and TC in HFD-fed mice exhibited a significantly elevated relative to the normal control group. The HFD+ARP, HFD+EX, and HFD+ARP+EX group sufficiently decreased the TG content, however, only the HFD+ARP+EX group could alleviate the abnormality in the TC content. In addition, the combination of ARP and EX exhibited an even greater diminishing relative than either ARP or EX alone, which was further confirmed by histology staining in the livers. As a major contributing factor to lipid biosynthesis, the genes involved in the fatty acid synthesis (FAS and ACC1) exhibited a significant higher expression in obese mice than those in normal control mice, leading to lipid accumulation (47, 48). In this study, the results showed a similar result that HFD feeding markedly elevated the protein expressions of FAS and ACC1, and these abnormal changes were efficiently suppressed by ARP, EX, or ARP+EX treatment group, which would contribute to decrease the lipid accumulation in the livers. In this regard, the data demonstrated for the first time that ARP combined with EX exhibited the interesting synergetic benefits through downregulating the fatty acid pathway, and thus improved obesity-associated abnormalities.

Apart from the upregulation of lipogenesis, the pathogenesis of MetS is usually characterized by chronic inflammation and oxidative stress (49, 50). Previous study reported that intake of ARP remarkably suppressed the secretion of TNF-α and IL-6, and their mRNA level (51). Consistent with the previous study, the results showed that ARP, EX, and ARP combined with EX during the HFD feeding could sufficiently attenuate the inflammatory response, including CRP, TNF-α, and IL-6, suggesting that the combination of ARP and EX exerted the beneficial effects on the obesity-induced inflammatory activity in obesity-induced mice. In addition, the combination of ARP and EX intervention significantly ameliorated diet-induced oxidative stress through increasing the antioxidant enzyme activity of GPX, SOD, and GST, and decreasing the content of MDA and protein carbonyl, which is consistent with the previous studies (51). Importantly, either ARP or EX treatment had no obvious effects on the liver ROS content, while the combination of ARP and EX could efficiently diminish the liver ROS level, and further maintain various cellular functions. These findings clearly indicated that the combination significantly protected livers against the obesity-induced oxidative injury, exhibiting a more sufficient effect than either ARP or EX group. As a critical transcription factor of phase II enzymes, Nrf2 modulates a protective effect in response to oxidative stress through its downstream genes, particularly NQO1 and HO-1 (52, 53). Here, the results found that combined ARP with EX markedly enhanced Nrf2-mediated phase II enzyme system than either ARP or EX treatment. From this, it was indicated that the combination of ARP and EX sufficiently attenuated the obesity-induced inflammation and oxidative stress through Nrf2 signaling pathway.

Mitochondria exerts crucial roles on energy expenditure and ROS production. Their dysfunction affects not only fat homeostasis but also ROS and cytokines levels, which further induces oxidative stress and chronic inflammation and contributes to the progression of MetS (54, 55). Therefore, the effects of the combination intervention involved in mitochondria was further assessed in HFD-fed mice. Here, it was shown that HFD feeding markedly suppressed the protein expression of mitochondrial complex subunits IV and V, and simultaneously suppressed the production of ATP in the livers. Interestingly, the combination of ARP and EX efficiently reversed these abnormalities and exhibited a greater synergetic effect than either ARP or EX treatment. Mitochondrial biogenesis is a complex process, mediated primarily the activation of PGC-1α (56). Growing evidence indicated that PGC-1α is a transcriptional coactivator and presently exerts a crucial node connecting metabolic regulation, redox control and inflammatory pathways (57, 58). Here, the results clearly showed that the expression of PGC-1α of the combination intervention showed a more markedly elevation than either that of ARP or EX treatment, suggesting a greater improvement of mitochondria. As noted above, the abnormality of lipid accumulation and oxidative stress could be the result of a deficiency in fatty acid oxidative and antioxidant defense related to mitochondrial dysfunction. Based on these results, it was speculated that the combination of ARP and EX strongly alleviated diet-induced metabolic disorders by improving mitochondrial function in a PGC-1α-dependent mechanism.

In conclusion, the current research demonstrated that combined ARP with EX intervention could protect livers in response to obesity-induced metabolic disorders by upregulating the PGC-1α-mediated mitochondrial biogenesis. Compared to either ARP or EX treatment, this combination exhibited greater effective synergetic benefits through reducing lipid accumulation, dampening inflammation, oxidative stress, and improving mitochondrial function in the livers. Together, these findings provide an additional evidence for the use of a combination of diet and exercise might be a feasible non-pharmacological therapy intervention for ameliorating obesity-induced metabolic disorders.

## Data Availability Statement

The original contributions presented in the study are included in the article/supplementary material, further inquiries can be directed to the corresponding author/s.

## Ethics Statement

The animal study was reviewed and approved by Animal Ethics Committee in Fujian University of Traditional Chinese Medicine and performed according to the guidelines of animal care and use for scientific purposes.

## Author Contributions

CC and XH: project administration. CC, MK, and QW: data curation. QX, WL, MY, and SL: investigation. CC, QW, QX, and WL: data analysis. CC, XH, and JT: writing. All authors have read and agreed to the published version of the manuscript.

## Funding

This study was supported by grants from the Scientific Research Foundation for the High-level Talents, Fujian University of Traditional Chinese Medicine (X2020001-talents).

## Conflict of Interest

The authors declare that the research was conducted in the absence of any commercial or financial relationships that could be construed as a potential conflict of interest.

## Publisher's Note

All claims expressed in this article are solely those of the authors and do not necessarily represent those of their affiliated organizations, or those of the publisher, the editors and the reviewers. Any product that may be evaluated in this article, or claim that may be made by its manufacturer, is not guaranteed or endorsed by the publisher.
